# RaveGuard: A Noise Monitoring Platform Using Low-End Microphones and Machine Learning

**DOI:** 10.3390/s20195583

**Published:** 2020-09-29

**Authors:** Lorenzo Monti, Mattia Vincenzi, Silvia Mirri, Giovanni Pau, Paola Salomoni

**Affiliations:** 1 Department of Computer Science and Engineering, University of Bologna, Mura Anteo Zamboni 7, 40126 Bologna, Italy; lorenzo.monti20@unibo.it (L.M.); giovanni.pau@unibo.it (G.P.); paola.salomoni@unibo.it (P.S.); 2 Master Degree in Computer Science, Department of Informatics, Systems and Communication, University of Milan—Bicocca, 20125 Milan, Italy; m.vincenzi14@campus.unimib.it; 3 Computer Science Department, University of California—Los Angeles (UCLA), Los Angeles, CA 90095-1596, USA; 4 School of Applied Sciences, Macao Polytechnic Institute, Macao, China

**Keywords:** noise pollution monitoring, edge computing, Internet of Things, smart city, artificial intelligence, machine learning, human-centric society

## Abstract

Urban noise is one of the most serious and underestimated environmental problems. According to the World Health Organization, noise pollution from traffic and other human activities, negatively impact the population health and life quality. Monitoring noise usually requires the use of professional and expensive instruments, called phonometers, able to accurately measure sound pressure levels. In many cases, phonometers are human-operated; therefore, periodic fine-granularity city-wide measurements are expensive. Recent advances in the Internet of Things (IoT) offer a window of opportunities for low-cost autonomous sound pressure meters. Such devices and platforms could enable fine time–space noise measurements throughout a city. Unfortunately, low-cost sound pressure sensors are inaccurate when compared with phonometers, experiencing a high variability in the measurements. In this paper, we present RaveGuard, an unmanned noise monitoring platform that exploits artificial intelligence strategies to improve the accuracy of low-cost devices. RaveGuard was initially deployed together with a professional phonometer for over two months in downtown Bologna, Italy, with the aim of collecting a large amount of precise noise pollution samples. The resulting datasets have been instrumental in designing InspectNoise, a library that can be exploited by IoT platforms, without the need of expensive phonometers, but obtaining a similar precision. In particular, we have applied supervised learning algorithms (adequately trained with our datasets) to reduce the accuracy gap between the professional phonometer and an IoT platform equipped with low-end devices and sensors. Results show that RaveGuard, combined with the InspectNoise library, achieves a 2.24% relative error compared to professional instruments, thus enabling low-cost unmanned city-wide noise monitoring.

## 1. Introduction

Noise can be defined as an unwanted sound or a sound that is loud, harmful and annoying to the ear. Noise pollution has been known since the ancient Roman time [1], where ironed wheels of wagons were battering the stones on the pavement, causing disruption of sleep and annoyance. It could be a source of stress for many people and, if it persists for long periods, it can cause numerous negative effects on health [2]. In fact, it is ascertained how this issue can cause problems on a personal level, including the decrease in cognitive abilities, such as a lower attention with the consequent cutback on working skills [3,4,5].

The causes of noise pollution are attributable to population growth, urbanization and the growth associated with the use of increasingly more powerful and varied noise sources, including means of transports (motorways, rails, and air traffic), which are considered as one of the main sources of the environmental noise [6,7]. In particular, given the increasing spread of urbanization and roads, rails, and air traffic, the problem of noise pollution is constantly expanding [8], producing negative effects from different points of view, affecting social, economic and work aspects [9,10], thus entailing a wide range of extra-auditory disturbances. In fact, according to the current literature, railway traffic [11,12] represents the second most impacting noise source affecting human modern life style [13], after road traffic [14,15,16,17], but before airports [18,19], industries and wind turbines [20,21], and port activities [22,23,24]. While sleep disorders with awakenings [25], learning impairment [26], hypertension ischemic heart disease [27,28], and especially annoyance [29] are recognized as the most common negative health effects related to prolonged exposition to noise pollution.

In this context, it is crucial having precise data on noise exposure levels. Nowadays, noise measurements in urban areas are mainly performed by designated organisations, gathering data in one or more points of interest around the city. This way, it is possible to collect and store data for later analysis, by using expensive devices such as sound level meters. However, this collection method (using an expensive equipment and requiring a human manual intervention) does not scale, as the demand for higher granularity of noise measurements in both time and space increases. A different perspective to mitigate this problem is to use an innovative approach relating to the interoperability of smart objects and their ability to access and retrieve data via the Internet, by applying the Internet of Things (IoT) paradigm [30,31,32,33]. Thanks to the ever growing spread of low-cost and increasingly computationally efficient devices, it is possible to exploit artificial intelligence strategies (for instance, adopting machine learning algorithms) to compensate for the gap in terms of accuracy that affects low-cost sensors and devices. Moreover, the use of IoT is even more and more adopted to monitor different conditions, both in indoor [34,35,36] and in outdoor scenarios [37,38].

In this work, we propose an IoT platform, named RaveGuard, aimed to monitor noise pollution, based on low-cost sensors and devices, adequately trained and instructed by means of machine learning algorithms. In RaveGuard, the physical devices used for monitoring noise are microphones. There are various types, distinguished according to their operations, and for each type, there are various models, with different characteristics and price ranges. Obviously, models with very different prices indicate different measurement accuracy in terms of decibels (dB). Therefore, the purpose of our research is to obtain a performance (in terms of accuracy in measuring noise intensity) similar to the one reported by the reference measurement instrument, even using low-cost microphones. In particular, in acoustic, such a reference measurement instrument is the sound level meter (phonometer). With our study, we aim at overcoming the precision gap between a low-cost microphone and a sound level meter calibrated. In order to reach this goal, we collected noise pollution samples by means of our IoT proposed platform, together with a professional phonometer, for two months, within the Campus of the University of Bologna. This allows us to create samples datasets, we have used to train different machine learning algorithms, with the aim of evaluating the one providing more accurate data, similar to the ones provided by the phonometer. Then, the most accurate machine learning algorithm has been exploited to create a library, named InspectNoise, that can be used by similar IoT platforms (equipped with low-cost microphones), supporting them in performing accurate noise pollution monitoring activities, in terms of sensing decibels. Another interesting point of attention is selecting the most appropriate dataset for adequately training a system by means of artificial intelligence strategies, as discussed in [39] and in [40]. In fact, in order to improve the quality of our training methodology, we have included also environmental data (such as temperature, humidity, air pollutant agents, and so on, collected by means of a multi-sensor station). Thus, we have obtained three different datasets (without environmental data, including dense environmental data, and including sparse environmental data) and we have applied four regressor models (choosing them among the most largely used in the literature). The training and the calibration activities have reported better results in terms of accuracy by using the second dataset, hence the one that includes also dense environmental data, showing an average error of 2.52 dB (against 5.20 dB, obtained without any artificial intelligence strategy to train the system).

The most innovative contributions of our study can be summarized as the following ones: (i) we have defined and proposed a low-cost IoT platform to monitor noise pollution; (ii) we have exploited artificial intelligence strategies to improve the accuracy of such a low-cost platform, making it comparable with the results obtained with professional and expensive equipment; (iii) we have added environmental data in the monitoring activities, enhancing the precision of the whole system in detecting noise pollution; finally, (iv) we have created three different datasets, on the basis of sampling activities last for 2 months, releasing them as available to the public audience.

This paper is organized as follows: Section 2 reviews and discusses related works, comparing approaches in literature with the one we propose. Section 3 presents an overview of our IoT platform and of the InspectNoise library, describing their workflows. Section 4 describes the hardware equipment and the sensors deployment of our RaveGuard, presenting how we collected our datasets and comparing costs with a professional equipment. Section 5 illustrates the methodology of the feature-based machine learning techniques we have exploited and the different datasets generated to improve monitoring of noise pollution, enhancing the accuracy of low-cost devices. Section 6 introduces the experiments, ground truth, and performance metrics, reporting their results. Finally, Section 7 discusses the results obtained by experiments we carried on and it concludes the paper by presenting some limitations and future works.

## 2. Related Works

The growing popularity of the IoT devices with significant computational power, the ubiquitous access to Internet connectivity and a huge quantity of low-cost sensors opens the door to a wide range of new applications [41]. In this perspective, it is therefore possible measuring the real impact of noise pollution through low-cost devices and microphones introducing a cheap, but powerful Wireless sensor network (WSN) platform that is readily available and widely deployed. Hereafter, we present the state-of-the-art in the area of environmental sensing, with a focus on noise pollution monitoring. In literature, we can find many works that we can categorize into three large groups that we will describe below.

### 2.1. Approaches Based on Propagation Models

Environmental noise is characterized as an abnormal and non-continuous phenomenon, and its intensity level changes rapidly over time. For this reason, countries such as European nations [42], United States, and Japan have developed their own sound propagation models so as to create noise maps by extrapolating local measurements to wider areas, showing the spatial distribution of noise exposure levels. The study described in [43] presents a field measurement approach that has been used in Karachi (Pakistan) to obtain information related to changes in traffic patterns and noise levels. Furthermore, it is possible to employ these models to produce exposure levels and to evaluate their effects on human health. Ref. [44,45,46] exploit such propagation models with the aim of validating noise levels. A different approach is studied in [47]: the authors investigated the temporal and the spatial variability of traffic noise in the city of Toronto (Ontario, Canada), finding out that the variability of traffic-related noise was mainly related to the spatial dimension instead of the temporal one.

### 2.2. Approaches Based on Mobile Crowdsourced Sensing

As already stated, prolonged exposure to noise can lead to various health risks, hence it would be strategic to densely monitor the noise, especially in the cities where traffic is constantly present [48]. Since the methods traditionally used for the detection of noise pollution use expensive and static equipment, they are not suitable for dynamic measurements [49]. Moreover, with the progress of technology and the spread of wearable devices, many avenues have been opened for the realization of applications for environmental monitoring [50]. Mobile Crowdsourced Sensing (MCS) is a human-centered detection paradigm, which allows citizens and tourists to provide data collected from their mobile devices, aggregate data collected in the cloud, extract knowledge and create awareness of some specific phenomena in the environment [51,52]. MCS represents a category of Smart Cities services that exploit citizens in the urban environment in order to collect and share data [53]. The large number of citizens and volunteers who adhere to these collection activities, as described in [54], could generate a huge amount of data and consequently increasingly accurate information, thus forming a more easily extensible architecture. The goal of MCS ecosystems is to inform citizens about the surrounding environment in real-time, so according to [51], users have a dynamic and always updated picture of the situation. In fact, smart mobility, together with the use of sensors for environmental monitoring and connectivity, can play a strategic role in providing useful information to citizens, so that they can improve their daily activities and the quality of their daily life [55]. These solutions are usually general enough to be used to monitor other kinds of environmental data, such as air pollution agents, contributing to the related analysis of fine particles. As an example, researchers from the University of Zhejiang, China, have developed a low cost sensor calibration method for air quality detection [56]. Furthermore, machine learning algorithms have been proposed in [57,58]: such algorithms aimed at better calibrating low-cost sensors while monitoring air pollution in IoT scenarios, so as to improve the quality of those data gathered by such a kind of low-cost devices.

### 2.3. Approaches Based on Wireless Sensor Networks

The last set of approaches that we present is the one based on Wireless Sensor Network (WSN). Such a kind of approach has been largely adopted in recent years, showing growing interest from the research community. An example is a system presented in [59], which lets monitor environmental noise by means of a WSN and users’ health status through a Body Access Network (BAN) sensor. Another interesting study is presented in [60], where the authors designed and developed specific noise sensors, placing them in fixed places in the city, so as to perform the monitoring activity, through a WSN. Moreover, the increased computing capacity of the nodes that create the network is allowing the addition of processing algorithms and artificial intelligence that provide more information the environment as exposed in [61].

Indeed, WSN is a technology applicable to a wide range of contexts related to IoT and monitoring activities, such as: environmental monitoring and urban sensing [62], natural disasters [63], healthcare [64], smart building [65], target objects tracking [66]. WSNs can be based on specific sets of low cost and low power nodes and sensors, connected to a gateway, letting the data collected within the network to reach an external communication network. An interesting key point is the cost sustainability: an detailed evaluation is necessary to identify if traditional or similar approaches are cheaper on a large scale.

## 3. RaveGuard: A System Overview

This Section presents an overview of RaveGuard IoT plaform, by describing (i) the system architecture (Section 3.1) and its main components devoted to sense noise and environmental data; and (ii) the main issues and workflow of the InspectNoise library (Section 3.2), we have designed and developed after the training phase (as described in the following Section 5), where we have chosen the machine learning algorithm and the dataset which provide the best result in terms of accuracy, with the aim of overcoming the gap between a professional equipment (phonometer) and a low-cost microphone, hence letting an IoT platform monitor noise pollution with high precision. The combined use of our IoT platform, equipped with a low-cost microphone, together with the InspectNoise library, will let us monitor noise pollution with a limited error in terms of sensed dBs, as described in the rest of the manuscript.

### 3.1. Our Platform Architecture

**Figure 1:**
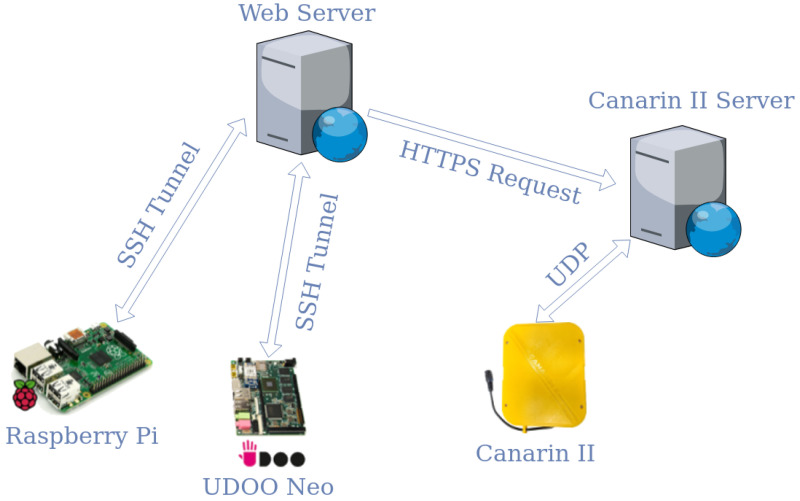
RaveGuard experimental architecture.

Figure 1 illustrates an overview of the IoT platform architecture we propose, which employed two sensing systems. One is aimed to noise pollution sensing, that takes over dB SPL (decibel Sound Pressure Level), based on a Raspberry Pi 2 model B and a low-cost microphone (a USB condenser microphone). In the data collection phase, we have equipped our RaveGuard platform with an UDOO Neo embedded device and a calibrated phonometer too (as depicted in Figure 1). That has been necessary to gather noise samples from both a professional equipment and a low-cost one. In particular, the calibrated sound level meter (on the UDOO New embedded device) represents the reference sound level meter (RSlm), while the USB condenser microphone (on the Raspberry Pi 2 mobel B) represents the device to be calibrated (DtbC). After the calibration phase and the training phase (exploiting artificial intelligence strategies), our IoT platform could perform noise pollution monitoring with just the low-cost sensors, avoding the use of expensive and professional equipment.

The second component is an environmental sensing system that can monitor temperature, relative humidity, air pressure and Particulate Matter (PM), in particular PM 1.0, 2.5 and 10, based on a Canarin II multi-sensor platform [55]. This is a crucial component to gather additional environmental data and include them in the training activities, during which we can evaluate and quantify how such information can improve the precision of the whole noise sensing platform, with the aim of reaching our goal: overcoming the precision gap between a low-cost microphone and a sound level meter calibrated. A detailed description of the whole hardware equipment we exploited in our proposed platform will be provided in Section 4.

In a first phase of our study, these two different components of our IoT platform architecture were meant to gather noise samples so as to create datasets, that will be exploited to train the whole system, by using the most performing machine learning algorithm. In particular, our RaveGuard collected data in different ways, generating three different datasets:

- Tiny dataset: this dataset is generated by the noise samples collected by the USB condenser microphone (DtbC) and by the calibrated sound level meter (RSlm).
- Full-sparse dataset: this dataset is generated by the noise samples collected by the USB condenser microphone and by the calibrated sound level meter, enriched with the environmental data gathered by the Canarin II multi-sensor station. Downsampling techniques have been applied on the noise pollution data in order to obtain the same sampling frequency of the Canarin II multi-sensor kit (1 per minute, against 1 per second of the noise monitoring component of our platform), this is why this dataset is considered sparse, when compared with the following dataset.
- Full-dense dataset: this dataset is generated by the noise samples collected by the USB condenser microphone and by the calibrated sound level meter, enriched with the environmental data gathered by the Canarin II multi-sensor station. In this case, upsampling techniques have been applied on environmental data collected by the Canarin II multi-sensor station, so as to obtain 60 occurrences per minute (using linear interpolation).

In generating these two latter datasets (full-sparse and full-dense datasets), downsampling and upsampling techniques have been necessary, because the data sampling frequency of the two architectural components is different: the noise monitoring component performs a sampling frequency of 1 per second, while the environmental monitoring component performs a sampling frequency of 1 per minute. Such sampling frequencies have been affected by the hardware equipment we have exploited in the experiment we have performed, as detailed in Section 4. In particular, the phonometer we have used allows a discrete sampling with 1 sample per second as sampling frequency, while the PM sensor within the Canarin II multi-sensor station generates one sample per minute, requiring time-consuming and resource consuming operations (in terms of computation). Hence, downsampling and upsampling techniques allow us to create uniform datasets, aligning sensed data over time. In general, our InspectNoise library and the whole RaveGuard system can perform and compute different sampling frequencies, with different rates (until 44,100 samples per second), on the basis of the capabilities of the hardware equipment actually used for the monitoring activities.

All datasets are open and available (https://github.com/LorenzoMonti/inspectNoise/tree/master/dataset).

Our three datasets has been then employed to define two models that can be found within the InspectNoise library and they have been then instrumental for the training phase.

**Figure 2:**
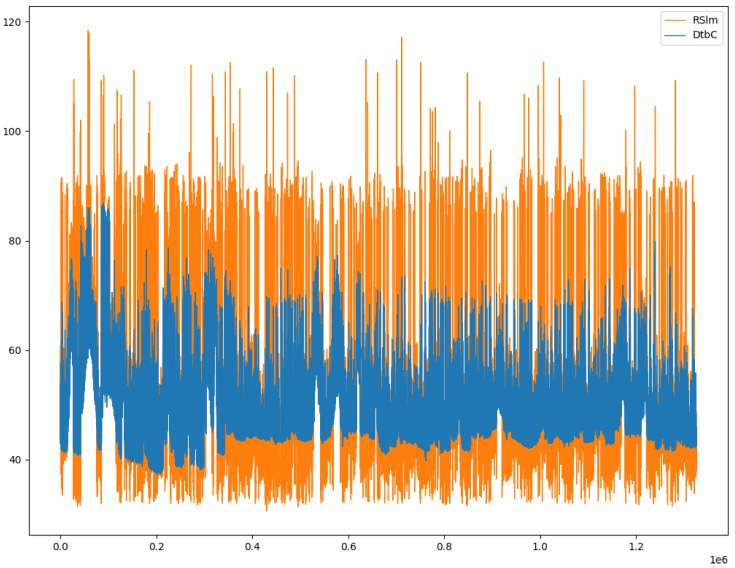
The amount of device to be calibrated (DtbC) and reference sound level meter (RSlm) data collected. The samples are aligned over time.

Such a training phase is necessary, because, after adding a correction equal to the average of differences between the errors, the difference in terms of dB SPL (decibel Sound Pressure Level) between DtbC data and RSlm data is 5.20 dB, as depicted in Figure 2. In fact, such a Figure represents the sound level samples we have gathered during two months, comparing the noise levels monitored by our low-cost microphone and the professional phonometer we have exploited during the data collection phase, during which we have observed an average error of 5.20 dB. Hence, it is interesting investigating and evaluating how the adoption of some artificial intelligence strategies (such machine learning techniques and methodologies) to train the low-cost version of the IoT platform, could bridge the accuracy gap of low-cost sensors and professional equipment results.

Regarding the network topology, our prototype (and the experiments we carried out) refers to a topology known in the literature as a star topology, where there is a single central node known as a hub or switch, while each other node of the network is connected to such a hub.

### 3.2. InspectNoise Library

In our study, we have designed and implemented the inspectNoise (https://github.com/LorenzoMonti/inspectNoise) library as the result of the datasets collection and of the machine learning training phase. The library takes into account two models: (i) the Tiny model, in which we analyze the USB condenser microphone dataset and the calibrated sound meter dataset; and (ii) the Full model, in which we analyze the datasets included in the tiny model, plus the dataset generated by the environmental data monitored by the Canarin II platform. The main goal of NoiseInspect is to support our RaveGuard platform, collecting and storing data and, once machine learning models were created, test them directly. Indeed, NoiseInspect has been trained using our three datasets and some machine learning algorithms, and it could be exploited by other similar IoT platforms, that could be equipped (using the full model) or not (using the tiny model) with environmental sensors.

This library has been developed in Python 3, exploiting in turn some libraries such as PyAudio and Pydub to manipulate input/output audio streaming Numpy in order to operate efficiently on audio data structures and pickle for serializing Python object structures. The system is basically in the off state and, when it is triggered, it will be brought to the on state. In particular, it is possible to set the number of seconds, which represents the duration of the noise monitoring activity.

**Figure 3:**
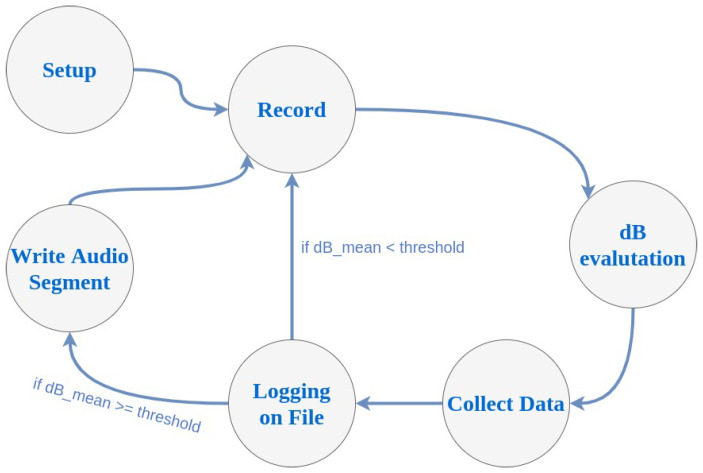
States of InspectNoise.

A schema representing the InspectNoise states (when the system is on) is depicted in Figure 3. At any time the system can return to the off state, when the sigterm is received, as well as after the preset operating time has elapsed. Figure 3 represents the behavior of the system, which is divided into six states:

1. Setup: In this state, the flags passed to the program are evaluated and the variables are prepared to perform the subsequent tasks.
2. Record: Ambient noise is monitored by creating audio segments.
3. dB evaluation The dB SPL for the previously detected audio segments are calculated.
4. Collect data: The data are compared with the previous ones to obtain at the end statistics as a minimum, maximum, and average.
5. Loggin on file: The dB value, obtained from the audio segments of the iteration, is written in the file, together with the timestamp identifying when the measurement took place.
6. Write audio segment: The audio segments are written in the memory, so as to be later exported in audio files.

## 4. Hardware Equipment and Deployment

This section is devoted to providing detailed information related to the RaveGuard hardware equipment and the sensors deployment. Moreover, a cost comparison between our low-cost platform and regular equipment devoted to noise monitoring can be found in Section 4.5.

In order to monitor environmental noise, our system has been equipped with a USB condenser microphone (DtbC), and a calibrated sound level meter (RSlm). They have been associated with an embedded device, having access to an Internet connection, so as to monitor the status of our platform. These devices have been configured to collect data and, independently, share them, in an automatic way, hence without the need of an explicit human intervention. This strategy was implemented to collect a sufficient amount of data, so as to implement proper machine learning strategies predicting the calibrated decibels of the RSlm. In this scenario, IoT devices have been used to facilitate and to automate the data retrieval and the sharing procedure.

In order to create a machine learning algorithm to calibrate low-cost microphones, a proper amount of data to train the machine learning models is necessary. First of all, a key point is the creation of datasets. As reported in [56,57,58,67], it is necessary to use a device that has to be calibrated, i.e., the microphone DtbC, and a device that provides reliable reference measurements, RSlm (taking into account the specific field of acoustics), so as to collect data that would create the datasets that will be used to instruct the machine learning models, as described in the following Section 5.

To ensure that the activity carried out by our platform can be monitored (without the need of a direct connection) and to form an architecture that supports maintenance activities, both such devices have been connected to embedded systems equipped with internet access. As already anticipated in Section 3.1, the devices used were: (i) a Raspberry Pi 2 model B with a low-cost USB condenser microphone, (ii) a UDOO Neo platform with a calibrated phonometer and lastly (iii) a low-cost environmental multi-sensor platform, named Canarin II. The following subsections provide a detailed description of all these devices.

### 4.1. Low-Cost Microphone and Raspberry Pi

In order to retrieve data from the USB microphone DtbC, we have used a Raspberry Pi 2 model B, a single-board CPU, an embedded system that incorporates on the same board a series of components, such as a microprocessor, a GPU, a RAM memory, a timer, a WiFi module, etc. The device is equipped with a series of analog and digital pins, so as to be able to interface with a wide range of sensors and equipment (available on the market). In fact, it has all the standard interfaces, such as USB, HDMI, etc. Thanks to these features, it is possible to connect the “Mini Akiro” microphone directly via the USB port. The WiFi module integrated in the card allows us to have an autonomous device that can share on the Internet all the data collected during the monitoring activities.

The InspectNoise library lets us monitor noise level in real time (acquiring noise in terms of decibels, dB) from any USB microphone connected to our platform. Such a library, developed in Python, it allows to save and store the samples taken in different files and formats. Sampling is carried out once per second, for a monitoring period that can be set from the command line. The microphone we have used in our prototype has been chosen for its availability on the market and its low cost. From the analysis of the data collected from the sound level meter and the microphone in Table 1, it seems clear that the values measured by the latter are not calibrated, since the difference between the values is very high.

The Raspberry Pi and the microphone therefore compose one of the edge-nodes of our platform, designed to monitor environmental noise.

### 4.2. Calibrated Phonometer and UDOO Neo

The UDOO Neo embedded has been devoted to obtaining the sound level meter data (RSlm) too, another single-board CPU device that incorporates all the components necessary to perform its tasks. This device is also equipped with the WiFi module, integrated into the board, which allows a simple connection with the other components of the architecture, so as to let exchange data. The sound level meter in question is the Uni-T UT351/352, a class 2 phonometer with a large range in terms of decibel (for our purpose), from 30 dB to 130 dB. Through the analog input of the board, we have connected the sound level meter by capturing its voltage by jumpers. The sound level meter expresses 10 mV in output for each decibel measured in the input. Thanks to the hybrid architecture proposed by UDOO Neo, which contains both a microcontroller (Arduino-like) and a microprocessor (Raspberry-like), it was possible to acquire and save data via a microcontroller in a simple and compact way, managing the network and forwarding these data via such a microcontroller. This IoT device, consisting of the UDOO Neo and a calibrated sound level meter, compose the second node of our developed edge architecture. On the contrary of the node described in the previous subsection, this has been equipped with a sound level meter, that constitutes the reference device for calibration.

### 4.3. Canarin II

A peculiar characteristic of our RaveGuard platform, which represents a very innovative aspect of our proposed solution if compared with the other noise monitoring systems, is the inclusion of a multi-sensor platform devoted to collect environmental data.

In particular, in our prototype, we have exploited the Canarin II architecture, an in-house printed circuit board (PCB) hosts the board and the sensors welded, summing up the PCB and the battery fits in a 19 × 15 × 7 cm, weighting about 900 gr. The PCB is contained in a in-house 3D printed PLA box, which has been designed to wrap the battery too. Such a platform has been developed and used for research purposes thanks to the synergistic collaboration among University of Bologna, Macao Polytechnic Institute, the Asian Institute of Technology, and the Pierre and Marie Curie Sorbonne University. This multi-sensor station is equipped with different sensors, such as environment sensors to sense air contaminants, gathering formaldehyde, PM1.0, PM2.5, and PM10 values. In addition, temperature, relative humidity and air pressure sensors are included in such a station. The communication between the station and our Web server is managed by a WiFi module that allows the device to act as a node in our infrastructure. During the data acquisition phase, the platform was placed alongside the other embedded devices in order to obtain a different kind of data, so as to have a better calibration level. Therefore, the measures of the environmental components collected by our Canarin II include: temperature, relative humidity, PM10, PM1.0, PM2.5, and pressure. A more detailed description of the Canarin II platform, its characteristics and performances can be found in [55] and in [68].

### 4.4. Sensors Deployment

All the nodes described in the previous subsections compose the entire RaveGuard platform architecture, as shown in Figure 1. In this subsection, we are going to present at a higher level of abstraction, illustrating the sensors deployment. First of all, the nodes used to acquiring the data relating to noise pollution have been positioned: the first consists of the Microphone-Raspberry Pi, while the second consists of the Phonometer-UDOO neo. Both devices were placed close together and at the same height, so that they performed equally and were subject to the same conditions. Each of them uses the reverse SSH tunneling technique (as depicted in Figure 1), so as to be able to connect to our server (placed within the university campus) to communicate, be reachable, and upload data. The samplings were performed by the devices with a frequency of one sample per second, for a period of about 12 h a day, therefore having 43,000 occurrences available every day. For a period of about 60 days, more than two million records make up the dataset. The Canarin II platform was also placed close to the microphone and the sound level meter to capture environmental data and thus achieve optimal calibration levels. In this case, such a multi-sensor station generate data with a frequency of one sample per minute. This is mainly due to the PM sensor, which requires a specific computational time. Gathered data are then forwarded via UDP protocol and stored on an ad-hoc server. This way, it is possible to query our server in order to interact and access all the necessary environmental data.

The entire monitoring process was carried out regularly for a period of about two months, with daily sampling lasting about twelve hours. At the end of the process, the data collected by the phonometer and the microphone have been transferred to the server, by using the existing SSH tunnel and through HTTPS request for the Canarin II platform. It is worth mentioning that such a technique is not mandatory to let our platform work: it has been necessary to overcome some connectivity limitations we experienced in the geographic location where we placed our prototype to collect the data for our experiment. Actually, once we have gathered the samples we needed to create the datasets, our RaveGuard platform can be used with similar hardware equipment, with other IoT-standard protocols, with other networks, and with other connectivity capabilities. The same is for our NoiseInspection library, which could be exploited in IoT contexts.

Hence, thanks to such a sensors deployment for this specific experiment, it was possible to create a dataset large enough to perform the machine learning strategies, as described in the following Section 5.

### 4.5. Costs Comparison

As we are going to describe in Section 2, several solutions already exist that provide noise pollution monitoring and sensing activities. One of the most interesting and innovative aspects of the platform we propose is the exploitation of environmental data (to collect more and different information to instruct the machine learning models) and the low cost of the hardware equipment, with performances and efficacy that can be compared with a professional and expensive equipment.

In Table 2, we report the costs related to the sensors and the components that compose our platform and the costs of a professional out of the box solution, so as to compare them.

The sensing platform we propose is an open-source, low-cost and intelligent solution (thanks to the machine learning models we have adopted to train the system), which can real-time and continuously (24/7) monitor noise pollution. In particular, our RaveGuard is a scalable solution, because all the computation activities take place on the device; moreover, exploiting embedded devices, our platform is efficient in terms of energy consumption too, if compared with other monitoring stations and units that are often used to gather environmental data. Indeed, this is not true if we compare the energy consumption required by the Raspberry Pi 2 board with other IoT devices. In fact, Raspberry Pi 2 is less effective in this sense than other similar devices [69].

## 5. Training RaveGuard: Our Methodology

In this section, we provide an overview of the methodology we have used to train our system. In fact, in order to improve the accuracy of low-cost microphones in our experiments, some machine learning algorithms have been exploited and examined for each dataset. In particular, we took advantage of four of the most widely used regressor models in the literature (linear regression, logistic regression, random forest and support vector regression), as described in the following subsections.

### 5.1. Linear Regression

Linear regression is a basic and commonly used type of predictive analysis. It predicts the relationship between two variables such as in the case (i) tiny dataset, in which we have only the microphone samples that represent the independent input variable *X*, while the calibrated values collected by the sound level meter the dependent one, to be predicted in output *Y* as show in Table 1 and the model in Equation (1).

(1) $$y_{reference}\left(t\right)=\beta_{0}+\beta_{1}\times \left[dB_{microphone}\right]$$

A natural generalization of the simple linear regression model is a situation including the influence of more than one independent variable to the dependent variable, again with a linear relationship such as with the (ii) full dataset, that includes environmental data. This is quite similar to the simple linear regression model, but with multiple independent variables contributing to the dependent variable and therefore multiple coefficients for determining and complex calculation due to the added variables as show in Equation (2).

(2) $$Y_{reference}\left(t\right)=\beta_{0}+\beta_{1}X_{1}+\beta_{2}X_{2}+....+\beta_{n}X_{n}$$

### 5.2. Polynomial Regression

The polynomial models can be used in those situations where the relationship between study and explanatory variables is curvilinear and a linear regression is not enough. So, it is used to overcome underfitting problem, sometimes a nonlinear relationship in a small range of explanatory variables can also be modeled by polynomials and we need to increase the complexity of the model. As shown in (3), this is a linear model, because the coefficients/weights associated with the features are linear.

(3) $$Y_{reference}\left(t\right)=\beta_{0}+\beta_{1}x_{i}+\beta_{2}x_{i}^{2}+....+\beta_{n}x_{i}^{n}$$

A grid search has been prepared to test all polynomial values. Furthermore, to avoid overfitting, by increasing the degree of the polynomial, the Ridge Regression regulation was introduced to limit the theta values calculated by the algorithm, via an origin-centered hypersphere.

### 5.3. Random Forest

Random Forests is a learning model based on ensemble learning, so, in this case, different algorithms are combined to obtain a better prediction model, solving regression and classification problems [70]. In this case, the results obtained from different decision trees are combined, which are aggregated to form a forest. This algorithm extrapolates *N* random records from the dataset, to build a decision tree based on the latter. This procedure is repeated *N* times, so as to create *N* decision trees that cooperate in the regression or classification task. In brief, a random forest model consists of a large number of individual decision trees that work as an ensemble. Every decision tree in the random forest generates a class prediction and the class with most votes becomes the model prediction. It will be necessary to choose the minimum number of decision trees to build the forest, and each tree will be built using a bootstrapped random sample from the training set. Design of the decision tree required the choice of an attribute selection measure and a pruning method. Exists several approaches to select the attributes for the decision tree induction and most approaches assign a quality measure directly to the attribute. The most frequently used attribute selection a measures in decision tree are the Gini Index [71] and the Information Gain Ratio criterion [72]. The random forest classifier uses the Gini Index as an attribute selection measure, which measures the impurity of an attribute with respect to the classes. For a given training set *T* selecting a value at random and saying that it belongs to some class $C_{i}$, it’s possible write the Gini Index as:

$$\sum \sum_{j\neq i}(f\left(C_{i},T\right)/|T|)(f\left(C_{i},T\right)/\left|T|\right)$$

where f(Ci,T)/|T| is the probability that the selected case belongs to class $C_{i}$. Every time a tree is grown to the maximum depth on new training data using a combination of features. These fully grown trees are not pruned. As exposed by Quinlan [72], this is one of the major advantages of the random forest classifier over other decision tree methods. Therefore, the random forest method consists of *N* trees, in which *N* is the number of three, where the users can defined any values. To classify a new dataset, each case of the datasets is broadcasted to each of the *N* trees. In this case, the forest chooses a class having the most out of *N* votes.

### 5.4. Support Vector Regression

The SV algorithm is a nonlinear generalization of the Generalized Portrait algorithm developed in Russia in the sixties [73,74]. The support vector machine (SVM) in its present form was largely developed at AT&T Bell Laboratories by Vapnik and co-workers [75]. SV learning has now evolved into an active area of research and it is widely applied to various fields, such as regression estimation, pattern recognition, and probability density function estimation. It is an exclusively data based modeling technique with a powerful potential for function estimation application. It was originally developed for classification purposes (SVC), before being extended also to regression problems (SVR). More formally, a support vector machine constructs a hyperplane, or set of hyperplanes, in a much higher dimensional space, which can be used for classification or regression. The support vector regression method aims at finding optimum hyperplane using the max-margin idea and minimizing the training error between the training data and identified function by means of the loss function. SVR maximizes the margins around the separating hyperplane. The decision function is fully specified by a subset (usually small) of training examples, called support vectors. Intuitively, we can use the optimization of max-margin to reduce the number of weights that are nonzero to just a few that correspond to the important features in order to separate line. Moreover, to keep the computational load reasonable, the mapping used by SV algorithms are designed to guarantee that dot products of input data vectors must be processed in terms of variables in the original space using the kernel function k(x,y) selected to fit the problem.

## 6. Results

In this section, we are going to present the application of the previously described machine learning algorithms, on the basis of our three datasets, reporting and comparing the obtained results. In particular, with the phonometer collected data as ground truth, four error metrics were used to compare all combinations of algorithms and datasets. The error metrics include mean square error (MSE), relative error, $R^{2}$ coefficient and root mean squared error (RMSE). The following subsections report the results we have obtained for each dataset.

### 6.1. Performance of the Calibrated dB Starting from Microphone Ones (Tiny Dataset)

This first approach involves the creation of the simplest dataset (the so-called *tiny* one), consisting of the samples gathered by the microphone **DtbC** and the sound level meter **RSlm**, then used as a basis for the realization of the following methods. The dataset was divided into training set (2/3) and validation set (1/3), used respectively for training and model evaluation.

**Figure 4:**
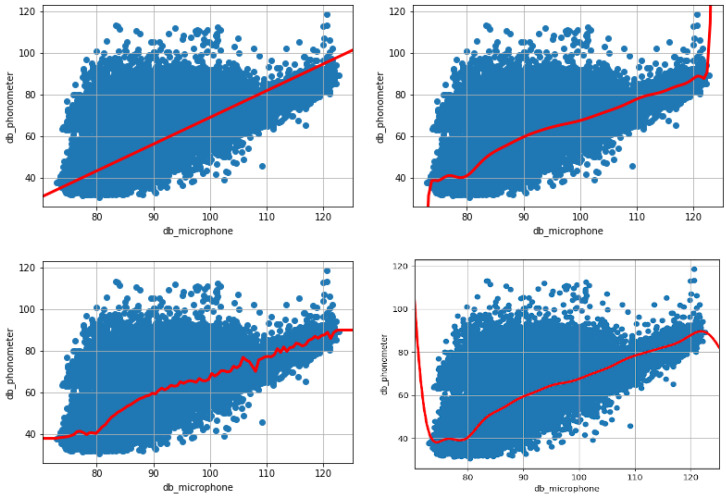
All the images show the correlation between the **DtbC** dB and **RSlm** dB. The top left image shows the univariate linear model, the top right image instead shows the polynomial model, the bottom left image the model based on Random forest and the bottom right image the model based on Support Vector Regression.

The linear regression method, as shown in Table 3 (first column), reports a value of 0.81 for the coefficient $R^{2}$, while the prediction shows an average error of 5 dB (Root Mean Square Error, RMSE). Finally, the relative error, which indicates the average error percentage of the predicted values compared to the real ones, is 7%. This linear model overlaps on the training set data, as depicted in Figure 4 (top left).

The best results obtained with polynomial regression, shown in Table 3 (second column), were obtained with a polynomial of degree 50, which is the maximum tested, and with 0.1, as the weight of the regularization. It is worth noting that, by continuing to increase the degree of the polynomial, the error did not undergo major changes. It is therefore not necessary to continue increasing the degree as higher computational resources would be needed to obtain a non-significant increase in terms of accuracy. This model approximates the variability of the data better than the previous one, since it has an $R^{2}$ coefficient of 0.85. Moreover, in the prediction of the calibrated decibels, it reports an average error of about 4.15 dB. Finally, the relative error is 5.77%. As Figure 4 (top right) shows, this polynomial model overlapped on the training set data.

The grid search technique was used for the random forest model, to search for the number of decision trees that would provide the greatest possible accuracy in prediction. Up to 140 decision trees have been tested. The best result was achieved precisely with 138 trees. The values of the model evaluation parameters are shown in Table 3 (third column). This model approximates the variability of the data as it has an $R^{2}$ coefficient of 0.85. Moreover, in predicting the calibrated decibels, it reports an average error of 4.16 dB, slightly higher than the previous model. Finally, the relative error is 5.77%. The Random Forest model overlapped on the training set data is depicted in Figure 4 (bottom left).

The Support Vector Regression method, as shown in Table 3 (fourth column), obtains a coefficient $R^{2}$ of 0.85. Moreover, the average error of the prediction is 4.19 dB. Finally, the relative error, which indicates the average error percentage of the predicted values compared to the real ones, is 5.75%. The Support Vector Regression model overlapped on the training set data is depicted in Figure 4 (bottom right).

### 6.2. Performance of the Calibrated dB Starting from the Microphone Ones and from the Canarin II Data, Using a Frequency Per Second (Full-Dense Dataset)

In this second approach, in order to predict the calibrated decibels, we have taken into account not only those ones measured by the microphone, but we have considered also the environmental data collected by Canarin II platform. The goal is to have a calibration precision more accurate than the one obtained with the previous method, by including the set of environmental data in the analysis. For the creation of the dataset, the data referred to **DtbC** and **RSlm** are the same used in the previous model, plus the environmental data.

The Canarin II station gathers data with a sampling rate of one minute, which leads to a number of occurrences of 59/60 lower than the sampling carried out by **DtbC** and **RSlm**. We decided to maintain the frequency used by the latter, which leads to the need to expand Canarin II data to obtain 60 occurrences per minute taking into account the 30 day period, thus obtaining a number of occurrences exceeding one million. *upsampling* techniques were applied from minute to second using the linear interpolation strategy. A fragment of this second dataset is reported in Table 4.

Also in this case, the models tested will be linear regression, polynomial regression, Random Forest and Support Vector Regression, and the results obtained in this second approach will be reported. The data were divided into training (2/3) and validation set (1/3). Unlike the previous case, the regression will be multivariate, i.e., the value of the dependent variable *Y* is estimated starting from the value of a set of independent variables *X*.

The first model tested, the linear regression model, shows the results as reported in Table 5 (first column). It is possible to notice that through the addition of new features relating to environmental data, the simple linear regression model leads to slightly better results than those ones obtained from the linear regression applied to the first dataset. Having a lower relative error and a higher $R^{2}$ coefficient, consequently also the average error resulted in each prediction (RMSE) is lower.

The polynomial regression model was tested and, as well as in the previous case, the grid search technique was used as a regularization technique, so as to avoid overfitting by increasing the degree of the polynomial. Polynomials up to grade eleven were tested, the latter also allowed to achieve the best result. The models have been tested with and without regularization, obtaining practically the same result. Table 5 (second column) shows the best results obtained with a three grade polynomial. It is interesting to note that such a model approximates the variability of the data very well, leading to an $R^{2}$ coefficient of 0.91 and an RMSE of 3.25 dB.

In the previous case, the Random Forest model has allowed, with a reasonable number of trees (100), to achieve the same results as the polynomial model with degree 20 polynomial. In Table 5 (third column), the results obtained by applying this machine learning model with this second dataset. The latter results show how the model best represents the variability of the data, obtaining an $R^{2}$ coefficient of 0.94, while the average error committed in the predictions (RMSE) is only 2.52 dB.

The gradient boosting model was also tested, also based on decision trees. In this case, each observation is weighted equivalent to the creation of the first tree. In this case, the weight associated with difficult to predict observations is increased, and vice versa, the weight of easily predicted observations is decreased. A second tree is then built on these new weighted data. Once this is done, the regression error made by joining these two trees is calculated to build a third tree. This procedure is performed iteratively, to decrease the residual error each time, for a number of times equal to those specified by its hyperparameter. Also in this case a grid search has been set up to search for the best value of the hyperparameters. The models were tested with a limited number of hyperparameters, as the results did not improve significantly. Up to 100 iterations (boosting stages) have been tested and the results obtained are those one reported in Table 5 (fourth column).

Regarding Support Vector Machine model, the results are shown in Table 5 (fifth column). The variability of the data expressed through $R^{2}$ coefficient is 0.92. Moreover, in predicting the calibrated decibels, an average error of 3 dB is obtained.

### 6.3. Performance of the Calibrated dB Starting from the Microphone and from the Canarin II Ones (Full-Sparse Dataset)

In this third approach, the acoustic data collected by the microphone and by the sound level meter, already prepared in the initial dataset, and those ones gathered by the Canarin II are used again. Also in this case, considering the same features of the previous one, multivariate regression is carried out with the aim of obtaining better results to perform the calibration. Despite the similarity to the previous approach, in this case we exploit the sampling frequency of the Canarin II (hence, one sample per minute, instead of one per second), as shown in Table 6, which reports a fragment of the third dataset. This means that, performing the sampling for 60 days, we collected about 40,000 occurrences, made available for a third dataset. Also in this case, we have based the experiments on the machine learning models tested in the previous subsections.

The first test, also in this case, is based on the linear regression model, whose results are presented in Table 7 (first column).

Regarding the polynomial regression model, also in this case, the best values of the hyperparameters are obtained by using the search grid technique. Having a much lower number of occurrences, it was possible to increase the degree of the polynomial, but failed to achieve better results than those ones obtained with the polynomial regression using the previous dataset. Table 7 (second column) shows the results obtained with degree 4 polynomial and regularization weight 11.

Since the number of samples was much lower than in the previous analysis, it is possible to test the Random Forest model with a greater number of decision trees (up to 300). The best results, shown in Table 7 (third column), are those ones obtained with 269 decision trees. Despite the radical increase in the number of decision trees involved in ensemble learning, it has not been possible to approach the precision achieved through the random forests applied to the previous (larger) dataset.

Lastly, the Support Vector Regression with gaussian kernel (that is shown in Table 7, fourth column), obtained the best result in this experiment. This model approximates the variability of the data as it has an $R^{2}$ coefficient of 0.90. Moreover, in predicting the calibrated decibels, the average error reported is of 3.42 dB.

All the results reported in these three experiments are reproducible and the code is located in the library repository inside the tests folder (https://github.com/LorenzoMonti/inspectNoise/tree/master/tests).

## 7. Discussion and Conclusions

The second experiment, described in the previous section, is the one that holds the best result in terms of accuracy. This is mainly due to the pre-processing of the dataset (that is the full-dense one). In fact, in addition to the very high number of occurrences (over one million), it also contained all the features relating to environmental conditions. By analyzing the models applied to this second dataset, it is worth noting that the best accuracy is achieved by the regression model based on the random forest. By using a limited number of decision trees (100) (to avoid a disproportionate increase in training times), it was possible to achieve very good results (see Table 8). In fact, the average error (in making predictions) is approximately 2.52 dB, with respect to the values measured by the reference device, i.e., the sound level meter. Furthermore, the model approximates the variability of the data in a very good way, having a coefficient $R^{2}$ of 0.94 and a relative error of 2.24%. Furthermore, it is important to highlight that such results should be compared with the RMSE error reported without the use of any type of machine learning technique, which is 5.20 dB (vs 2.52 dB obtained with our RaveGuard platform, adequately trained).

**Figure 5:**
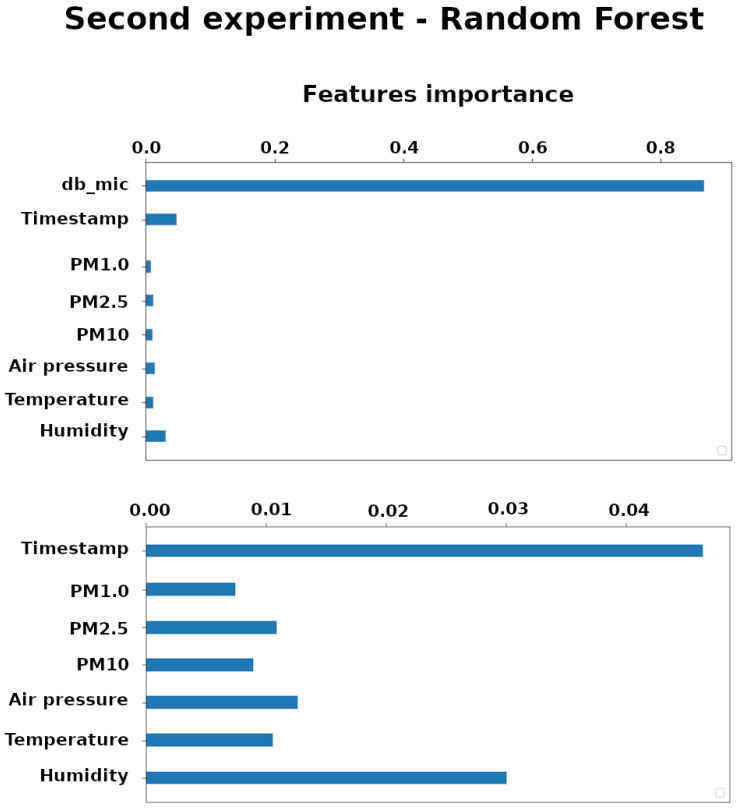
In the upper part are shown all the features of the second experiment dataset. The dB_mic feature has a greater importance. The dB_mic feature is omitted in the lower part to make the other features more readable.

Feature importance scores play an important role in a predictive modeling project, including providing insight into the data, insight into the model, and the basis for dimensionality reduction and feature selection that can improve the efficiency and effectiveness of a predictive model on the problem. For this reason, the scores of each feature taken into consideration for the experiments was calculated and shown in Figure 5. The chart presented on the upper side in the Figure 5 shows all the features of the experiment dataset. It shows how the feature that corresponds to the microphone data (dB_mic) has a greater importance (an order of magnitude more than the other features). In order to make the other features more readable, the dB_mic feature is omitted on the lower side of the figure, bringing out the Humidity and the Timestamp as other important features.

The ultimate goal of training these learning models is to export and integrate them into a real-time noise monitoring tool. Such a monitoring tool will be employed on embedded systems (Raspberry Pi 2), with the aim of sensing noise pollution within a University Campus, by means of a low cost microphone on our prototype (the one calibrated for the definition of the datasets). Once equipped with an algorithm that can monitor calibrated decibels, it will be possible to contribute to the detection of noise pollution in a more accurate way. Two different models have therefore been implemented for this purpose in our noise detection tool, which can be used via the command line. From the results obtained by the training phase of the learning models, we carry out two different calibrations, one simple (tiny) and one more complete (full), as described in the following:

- Tiny calibration: this type of calibration is designed to be as light and simple as possible, so that it can be carried out with the use of the least number of components. This calibration is based on the best calibration model obtained from the first method, which is the polynomial regression model (see Table 3, second column). It could be based, in an indifferent way, also on the model trained using the random forests, being the results almost identical. These models are based on the prediction of decibels calibrated starting only from the samples carried out by the microphone (univariate regression). This is why they do not require any additional component and the edge previously prepared for the construction of the dataset can be used directly. Once the decibel value has been obtained from the audio segment collected by the microphone, it is necessary to feed it to the model previously loaded from a file, so as to obtain the prediction of the calibrated decibels. These values, as shown in Table 3 (second column), will have an average error of 4.15 dB.
- Full calibration: this calibration aims to be more precise, hence requiring the use of a greater number of components. It can take advantage of the best tested model, hence the model based on random forests applied to the second dataset (see Section 6.2 Random Forest). This multivariate regression model bases its predictions on a set of independent variables (X) consisting of both the decibels detected by the microphone and the environmental data made available by Canarin II. By integrating this model into our noise monitoring tool, it is necessary to equip the Raspberry Pi (or more generally the embedded system used) with all those sensors useful for monitoring the environmental data necessary to obtain more accurate predictions. This is necessary because it is necessary to have all the information available on the same device, so as to provide a real-time sampling service. Once the decibel value has been obtained from the audio segment collected by the microphone, it is then necessary to input it to the model, together with all the other environmental data. Hence, it would therefore be necessary to equip the Raspberry Pi with sensors for: humidity, atmospheric pressure, temperature, PM1.0, PM10 and PM2.5. The model, once all this information has been received, predicts the decibels calibrated with an average error of 2.52 dB.

Hence, two different application methods have been proposed in our study, with the respective models applied, so that, depending on the resources available, it is possible to integrate the models (and the related accuracy and precision) in the system. The tiny calibration method available in the library (https://github.com/LorenzoMonti/inspectNoise/blob/master/calibration/model.bin), is simpler to be applied, as it does not require the purchase of additional components. On the other hand, if the sensors required to detect the aforementioned environmental data are available, then the full calibration method would allow us to achieve better precision.

In summary, we have presented RaveGuard, a noise pollution monitoring platform that exploits machine learning algorithms to improve the accuracy of low-cost microphones. Three different experiments were carried out, each of them with three different starting datasets. To collect the necessary data, an experimental setup consisting of three different nodes was deployed. The first node consists of a Raspberry Pi and a low-cost USB condenser microphone; the second node is composed of a UDOO Neo and a calibrated sound level meter, and, finally, the third node consists of a Canarin II platform, devoted to acquiring environmental data. The simplest dataset includes only the data of the low cost microphone and the calibrated sound level meter which acts as ground truth. The other datasets include environmental data, acquired and saved thanks to the Canarin II platform. Different regression models have been applied to each dataset, with the aim of mitigating the problem of the low accuracy of low cost devices (compared to the calibrated ones). The results achieved reduced the accuracy gap from an RMSE error of 5.20 dB to 2.52 dB, with our best model applied to the denser dataset, and to 4.15 dB, with our best model applied to the less dense dataset. Two different models have therefore been generated in our noise detection tool, which can be used via the command line. The first one (named tiny), is designed to be as light and simple as possible in order to be used with the minimum number of components, while the second one, that is more complete (named full), achieves the best accuracy.

It is worth mentioning that we did not take into account the various spectral characteristics of the microphone and of the professional sound level meter, because our main goal was to create datasets and a library to support monitoring activities, considering only the specific hardware equipment we exploited for our experiment, even if this could represent a limitation in our study. An interesting configuration and customization of our library and our platform could be planned and designed by considering such characteristics and by adapting the whole monitoring system on the basis of the specific microphone used for the sensing activities (i.e., distinguishing among low cost USB microphones and professional sound level meters).

We are currently working on the effective deployment of multiple instances within our University Campus to map noise pollution. Moreover, new models will be tested to further improve the system performance, on the basis of deep learning models, such as ANN or auto-encoder. We are confident that such models could improve the overall accuracy provided by the monitoring platform, but further investigations and comparisons have to be done. Moreover, together with the accuracy of the noise monitoring activities, it would be necessary to evaluate and then to find an appropriate balancing between the obtained accuracy and the corresponding efforts in terms of computation time and load of the proposed platform.

We are planning new experiments, collecting new data samples. This would allow us to apply adequate strategies to adequately collect and detect data from events related to the weather, such as rain and wind. In fact, in our experiment we did not acquire such kind of data, but they are fundamental in acoustic measurement, so as to properly clean the data from anomalous events. Indeed, this would significantly concur in improving the accuracy of our datasets and our InspectNoise library.

Moreover, the conduction of new experiments would give us the chance of better evaluating energy efficiency and scalability issues. Data about the specific energy consumption could be obtained and accurate estimation could be observed and then predicted. The same is for considerations about the system power, which should be connected to the electricity grid for long periods, hence an evaluation involving pros and cons related to the positioning of the whole monitoring platform should be provided, improving the overall efficacy of the system. Regarding scalability, another issue that should be taken into account and evaluated is the data storage capabilities and the comparison among the proposed solution (which is edge computing based) and other different architectural solution (i.e., distributed ones). In particular, during our experiment and data collection activities, we did not experienced any problem in terms of storage capabilities. However, a comparison among edge computing solutions and distributed solutions can contribute to enriching the state of the art in this field.

## Figures and Tables

**Table 1:** Comparison of $dB_{SPL}$ measured by the microphone and the sound level meter.

Datetime	${\mathrm{dB}}_{\mathrm{microphone}}$	${\mathrm{dB}}_{\mathrm{phonometer}}$
19:00:07	48.07	52.12
19:00:08	46.03	50.59
19:00:09	47.15	49.14
19:00:10	47.90	49.70

**Table 2:** Costs comparison between our platform and a calibrated phonometer (devices used to retrieve data and create the dataset).

InspectNoise		Phonometer	
Raspberry Pi 2	∼33.00 euros	Uni-T UTI 351	∼288.00 euros
“Mini Akiro” USB microphone	∼15.00 euros	Power supply	∼7.00 euros
Power supply	∼7.00 euros		
microSD 8Gb	∼5.00 euros		
**Total**	∼60.00 euros	**Total**	∼295.00 euros

**Table 3:** First experiment: evaluation coefficients for linear regression, polynomial regression, random forest and Support Vector Machine with rbf kernel.

	Linear Reg	Polynomial Reg	Random Forest	SVR (rbf)
**MSE**	22.328	17.262	17.326	17.561
**Relative average error**	7.08692%	5.77911%	5.77192%	5.75414%
**$R^{2}$ coefficient**	0.81034	0.85337	0.85283	0.85083
**RMSE**	4.725	4.154	4.162	4.1906

**Table 4:** Exemplary part of the second dataset.

DateTime	${\mathrm{db}}_{\mathrm{microphone}}$	${\mathrm{db}}_{\mathrm{phonometer}}$	PM1.0	PM10	PM2.5	Pres	Temp	Hum
19:00:13	48.18	51.07	3.0	7.0	5.0	1007.7	21.9	59.2
19:04:19	46.80	48.90	3.0	6.0	5.0	1007.7	21.9	59.2
22:06:54	54.10	59.37	3.7	5.7	5.0	1009.0	22.7	57.2
06:58:10	63.98	69.93	3.0	5.0	5.0	1008.8	24.9	58.4

**Table 5:** Second experiment: evaluation coefficients for linear regression, polynomial regression, random forest, gradient boosting and Support Vector Machine with rbf kernel.

	Linear Reg	Polynomial Reg	Random Forest	Gradient Boosting	SVR (rbf)
**MSE**	19.942	10.574	6.3558	11.978	9.4129
**Relative error**	6.41215%	3.62220%	2.24996%	3.93945%	3.10973%
**$R^{2}$ coefficient**	0.83268	0.91128	0.94667	0.8995	0.92102
**RMSE**	4.465	3.251	2.521	4.011	3.0681

**Table 6:** Exemplary part of the third dataset.

DateTime	${\mathrm{db}}_{\mathrm{microphone}}$	${\mathrm{db}}_{\mathrm{phonometer}}$	PM1.0	PM10	PM2.5	Pres	Temp	Hum
19:07:22	47.40	52.69	4.0	7.0	6.0	1007.7	21.9	59.3
19:08:22	46.96	50.43	3.0	5.0	5.0	1007.7	21.8	59.3
19:09:24	44.40	45.68	3.0	6.0	5.0	1007.7	21.9	59.3
19:10:25	45.78	48.25	3.0	6.0	4.0	1007.7	21.8	59.2

**Table 7:** Third experiment: evaluation coefficients for linear regression, polynomial regression, random forest and Support Vector machine with rbf kernel.

	Linear Reg	Polynomial Reg	Random Forest	SVR (rbf)
**MSE**	20.226	12.216	13.073	11.76
**Relative error**	6.48700%	3.97385%	3.87727%	3.70047%
**$R^{2}$ coefficient**	0.83203	0.89855	0.89143	0.90234
**RMSE**	4.497	3.495	3.61	3.4292

**Table 8:** Best model for each experiment.

	Best Model	$R^{2}$	RMSE	Relative Error
**Experiment 1**	Polynomial regression	0.85	4.15	5.77%
**Experiment 2**	Random forest	0.94	2.52	2.24%
**Experiment 3**	Support Vector Machine	0.90	3.42	3.70%
